# Enhanced cervical cancer and HIV interventions reduce the disproportionate burden of cervical cancer cases among women living with HIV: A modeling analysis

**DOI:** 10.1371/journal.pone.0301997

**Published:** 2024-05-23

**Authors:** Cara J. Broshkevitch, Ruanne V. Barnabas, Gui Liu, Thesla Palanee-Phillips, Darcy White Rao

**Affiliations:** 1 Department of Epidemiology, University of North Carolina-Chapel Hill, Chapel Hill, NC, United States of America; 2 Division of Infectious Diseases, Massachusetts General Hospital, Boston, MA, United States of America; 3 Harvard Medical School, Boston, MA, United States of America; 4 Department of Global Health, University of Washington, Seattle, WA, United States of America; 5 Wits Reproductive Health and HIV Institute, Faculty of Health Sciences, School of Public Health, University of the Witwatersrand, Johannesburg, South Africa; 6 Department of Epidemiology, School of Public Health, University of Washington, Seattle, WA, United States of America; University of Jos Faculty of Medical Sciences, NIGERIA

## Abstract

**Introduction:**

Women living with HIV experience heightened risk of cervical cancer, and over 50% of cases in Southern Africa are attributed to HIV co-infection. Cervical cancer interventions tailored by HIV status delivered with HIV antiretroviral therapy (ART) for treatment can decrease cancer incidence, but impact on HIV-related disparities remains understudied.

**Methods:**

Using a dynamic model calibrated to KwaZulu-Natal, South Africa, we projected HIV prevalence, cervical cancer incidence, and proportion of cancer cases among women living with HIV between 2021–2071. Relative to the status quo of moderate intervention coverage, we modeled three additive scenarios: 1) ART scale-up only; 2) expanded human papillomavirus (HPV) vaccination, screening, and treatment; and 3) catch-up HPV vaccination and enhanced screening for women living with HIV.

**Results:**

Under the status quo, HIV prevalence among women aged 15+ decreased from a median of 35% [Uncertainty Range (UR): 26–42%] in 2021 to 25% [19–34%] in 2071. The proportion of cervical cancer cases that were women living with HIV declined from 73% [63–86%] to 58% [47–74%], but incidence remained 4.3-fold [3.3–5.7] that of women without HIV. ART scale-up reduced HIV prevalence in 2071, but increased the incidence rate ratio to 5.2 [3.7–7.3]. Disparities remained after expanding cancer interventions for all women (incidence rate ratio: 4.8 [3.6–7.6]), while additional catch-up HPV vaccination and screening for women living with HIV decreased the incidence rate ratio to 2.7 [1.9–3.4] in 2071.

**Conclusions:**

Tailored cervical cancer interventions for women living with HIV can counteract rising cancer incidence incurred by extended life expectancy on ART and reduce disparate cancer burden.

## Introduction

In Southern Africa, cervical cancer is the leading cause of female cancer death and the second most frequent female cancer [1]. Just over half (53.2%) of cervical cancer cases in Southern Africa are attributable to human immunodeficiency virus (HIV), compared to 4.9% globally [2]. South Africa, a country with 26.3% HIV prevalence among women aged 15–49 [3], is home to the largest absolute number of women with cervical cancer living with HIV, at approximately 8,220 cases [2]. Women living with HIV have an estimated six-fold higher risk of cervical cancer compared to those without [2]. Together with risk factors including smoking, high parity, and gaps in access to screening and treatment, HIV infection contributes to profound global disparities in cervical cancer [4]. Cervical cancer burden among women living with HIV must be reduced to decrease overall cervical cancer burden.

Highly effective interventions are available for prevention and treatment of both HIV and cervical cancer, but their combined impact on the disproportionate burden of cervical cancer cases among women living with HIV remains unclear. Studies show that HIV antiretroviral therapy (ART) can mitigate cervical cancer risk [5], but may increase cervical cancer cases in the short-term by extending life expectancy for women living with HIV [6, 7]. Particularly in South Africa, cervical cancer incidence among women living with HIV on ART remains high [8]. There is also evidence that performance characteristics of cervical cancer screening tests [9–11] and effectiveness of precancer treatments [12] differ by HIV status. These differences, along with the heightened risk of cervical cancer among women living with HIV, highlight the importance of cervical cancer prevention and treatment guidelines tailored by HIV status. Recent modeling studies predicted that increasing vaccination or screening frequency for women living with HIV can significantly decrease near-term cervical cancer incidence [6, 13, 14]. A comparative modeling study additionally demonstrated reduced relative cancer incidence among women living with HIV with focused interventions, but assumed idealized coverage levels of precancer and cancer treatment and 100% precancer treatment efficacy [15]. The impact of cervical cancer interventions tailored by HIV status on the disparity in cancer risk between women with and without HIV, and proportion of cervical cancer cases that are women living with HIV, have yet to be fully quantified.

In this study, we applied a mathematical model to estimate future cervical cancer incidence in women with and without HIV under four scenarios with varying assumptions for HIV and cervical cancer prevention and treatment. The model was calibrated to KwaZulu-Natal, South Africa, a region with an estimated 37.3% HIV prevalence among women aged 15–49 in 2016 [16] and moderate coverage of HIV and cervical cancer interventions (approximately 59% of persons aged 15–79 living with HIV on ART with viral suppression [3], 57% of girls aged 9–14 vaccinated against human papillomavirus (HPV) [17], and 48% of women screened once-per-lifetime for cervical cancer [16]). We evaluated the projected impact of each scenario on total HIV and cancer burden as well as on disparities in cancer incidence by HIV status. Our estimates of the proportion of cervical cancer cases expected to occur among women living with HIV are valuable to help public health agencies anticipate needs and most effectively distribute prevention and treatment resources to reduce national incidence rates.

## Methods

We used the DRIVE (Data-driven Recommendations for Interventions against Viral InfEction) model [13], a dynamic, compartmental model of HIV and HPV transmission and disease progression, to project cervical cancer and HIV outcomes for the province of KwaZulu-Natal, South Africa between the years 2001 and 2071. The model population was stratified by age, gender, sexual activity risk group, and infection and disease status. We model oncogenic HPV infection (high-risk HPV; hrHPV), grouped as either nonavalent vaccine-type hrHPV (types 16, 18, 31, 33, 45, 52, and 58) or non-vaccine type hrHPV (all other oncogenic types). HIV disease progression was tracked by CD4 cell count and HIV viral load. HIV infection increased an individual’s risk of HPV acquisition and progression to cervical cancer, with risks increasing inversely with CD4 count. With prior distributions of model parameters derived from observed data, we used an Approximate Bayesian Computation- Sequential Monte Carlo method to calibrate the model to observed population-level demographics and historical data on HIV-1 prevalence, HPV prevalence, cervical intraepithelial neoplasia (CIN) prevalence, and cervical cancer incidence. We validated the model to recent HIV-1 prevalence and incidence data (model details in Rao *et al*. [13] S1 Appendix.

Beginning in 2004, we modeled use of ART among individuals who achieved HIV viral suppression. The proportion of persons living with HIV who were on ART and virally suppressed followed empirical data from South Africa between 2004 and 2017. We assumed that 57% of girls aged nine years received two doses of the bivalent HPV vaccine beginning in 2014 [17]. In this historical phase of the model, cervical cancer screening occurred once-per-lifetime with cytology between ages 35–39 years and coverage scaled up linearly from 0% in 2000 to 48% in 2016 [16]. Persons with CIN2+ were referred for colposcopic evaluation with biopsy, and women with confirmed lesions or cancer were treated with large loop excision of the transformation zone (LLETZ) or hysterectomy, respectively [18]. High loss-to-follow-up resulted in low levels of effective treatment with this three-visit strategy [19]. Effective treatment was lower for women living with HIV, reflecting lower sensitivity and specificity with cytology [9, 10] and higher treatment failure (16–23%) compared to women without HIV (9%) [12] (historic assumptions in Rao *et al*. [13] Section II.e. in S1 Appendix.

For the period 2021 to 2071, we modeled four scenarios (Table 1). The status quo scenario assumed a switch from the bivalent (2v) to the nonavalent (9v) HPV vaccine in 2021, and that HPV vaccination, cervical cancer screening and treatment, and the proportion of individuals with viral suppression remained at current levels (“Baseline”). A second scenario assumed scale-up of ART to the UNAIDS 90-90-90 targets (72.9% of persons living with HIV virally suppressed) by 2030 (“ART scale-up only”). We additionally modeled a scenario with ART scale-up along with enhanced HPV vaccination and cervical cancer screening interventions provided regardless of HIV status (“Enhanced cervical cancer interventions”). In this scenario, 90% of girls aged 9–14 years were vaccinated with the 9vHPV vaccine, and women received twice-per-lifetime cervical cancer screening with an HPV DNA test between ages 35–39 and 45–49. Screening test performance and treatment efficacy differed by HIV status, with higher test sensitivity, lower test specificity, and higher treatment failure for women living with HIV compared to women without HIV. Screening coverage increased from 48% in 2021 to 70% in 2030, and to 90% by 2045. Women who screened positive for hrHPV could receive immediate treatment with thermal ablation, or they were referred for LLETZ or hysterectomy, as appropriate. The final scenario added to the enhanced cervical cancer intervention scenario by targeting additional vaccination and screening to women living with HIV (“Enhanced cervical cancer interventions for women living with HIV”). In this scenario, 50% of women living with HIV received catch-up 9vHPV vaccination between ages 15–24 years, and cervical cancer screening occurred every five years between ages 25–49 (approximating recommended 3-yearly screening) [18]. All scenarios assumed that voluntary male medical circumcision (VMMC) coverage increased until 2017 following observed trends, with no future increases. We assumed that men without HIV who received VMMC had decreased risk of HIV acquisition but no protection against HPV acquisition (future assumptions in Section II. in S1 Appendix).

**Table 1:** Cervical cancer and HIV intervention scenarios beginning in 2021.

Scenario	Intervention			
	ART	9v HPV vaccination	Cervical cancer screening	Cervical cancer treatment
**Baseline**	60% of women and 44% of men aged 10–79 living with HIV are on ART with viral suppression; no ART scale-up from 2017	57% coverage among girls aged 9–14	48% screening coverage using cytology once per lifetime	Three-visit algorithm: 72% return for colposcopy; 51% of those indicated for precancer treatment return for LLETZ and 40% of those with cancer return for hysterectomy
**ART scale-up only**	ART scaled-up to UNAIDS 90-90-90 targets by 2030			
**Enhanced cervical cancer interventions**		90% coverage among girls aged 9–14	HPV DNA testing twice per lifetime, scaled up from 48% to 70% coverage by 2030, and to 90% coverage by 2045	Single-visit algorithm: 95% of eligible women receive thermal ablation, 80% of those ineligible for ablation receive LLETZ, and 40% of women with cancer receive hysterectomy
**Enhanced cervical cancer interventions for women living with HIV**		*Enhanced cervical cancer interventions* + 50% coverage of catch-up vaccination among young women living with HIV aged 15–24	*Enhanced cervical cancer interventions* + HPV DNA testing every five years among women living with HIV	

We estimated crude cervical cancer incidence rates over time among women aged 15+, stratified by HIV status. To quantify disparities in cervical cancer burden, we calculated the rate ratio of cancer incidence among women with and without HIV. We also estimated the proportion of new cervical cancer cases in women aged 15+ that are women living with HIV over time, in relation to changes in HIV prevalence. We calculated the median and the range of model outcomes simulated using the 25 best-fitting parameter sets as the uncertainty range (UR).

Modeling was performed in MATLAB version 9.11.0 (R2021b) and data visualization was conducted in R version 4.3.1 (2023-06-16) [20, 21].

## Results

### Status quo HIV and cervical cancer prevention (baseline scenario)

**Fig 1:**
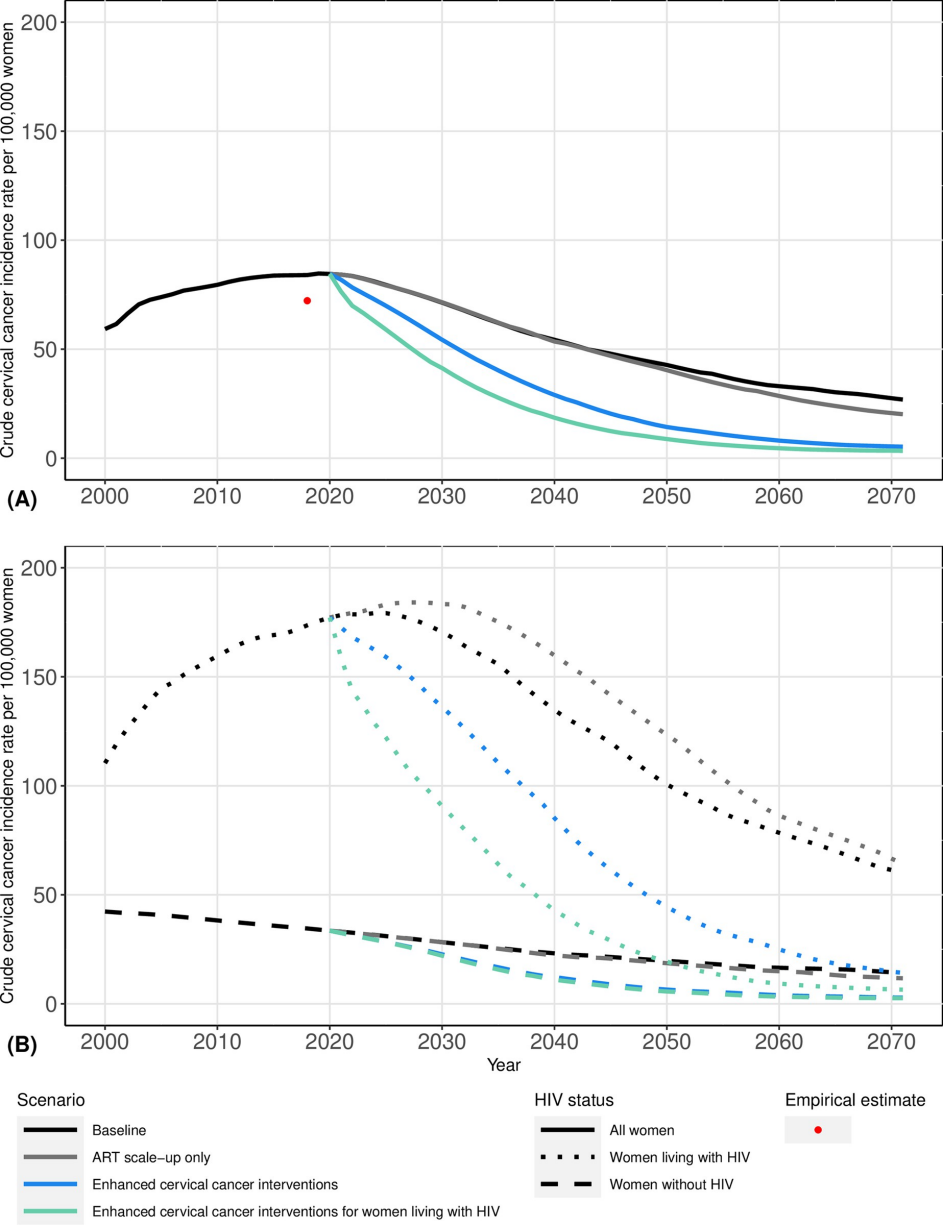
Median projected cervical cancer incidence rates per 100,000 women aged 15+ for the general population (A) and by HIV status (B) over time under simulated intervention scenarios. The data point in red was estimated by adjusting South Africa Globocan 2018 [1] rates by age to take into account higher HIV prevalence in KwaZulu-Natal, weighting rates by the median modeled population age distribution in 2018, and then summing to get an estimate of the cervical cancer incidence rate for all women aged 15+ in KwaZulu-Natal.

**Fig 2:**
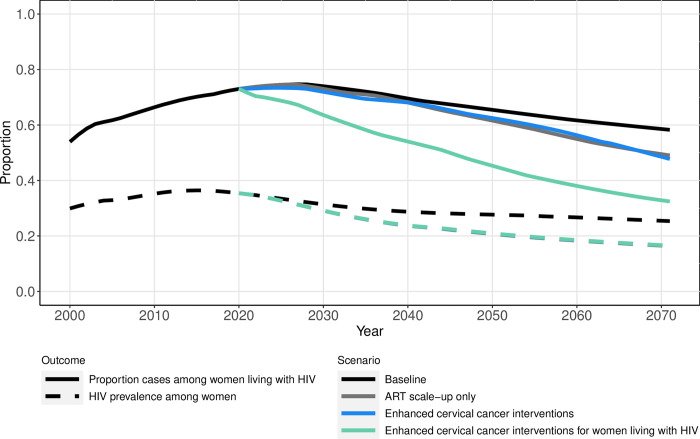
Median proportion of cervical cancer cases among women living with HIV aged 15+ and HIV prevalence among women aged 15+ over time under simulated intervention scenarios.

Our model reproduced observed increases in HIV prevalence and cervical cancer incidence since 2001 (Section III. in S1 Appendix). In 2021, HIV prevalence was 35% [UR: 26, 42%] among women aged 15+, and cervical cancer incidence was estimated at 84.2 [58.9, 123.3] cases per 100,000 women aged 15+ (Fig 1 and Table 2). Women living with HIV accounted for 73% [63, 86%] of cervical cancer cases in 2021 and experienced a cervical cancer incidence rate 5.6 [3.8, 9.1] times higher than women without HIV (Fig 2 and Table 2). With continuation of current intervention levels, we predicted that by 2071, HIV prevalence among women will decrease to 25% [19, 34%], and that cervical cancer incidence will decline to 26.9 [13.3, 53.3] cases per 100,000 women. Women living with HIV were predicted to represent 58% [47, 74%] of cervical cancer cases, with an incidence rate 4.3 [3.3, 5.7] times higher than that among women without HIV.

**Table 2:** Cervical cancer and HIV outcomes over time under simulated intervention scenarios with uncertainty ranges [URs].

Scenario	Year	Total cervical cancer incidence per 100,000 women *Median [UR]*	HIV prevalence among women *Median [UR]*	Proportion of cervical cancer cases that are women living with HIV *Median [UR]*	Rate ratio of cervical cancer incidence among women living with HIV compared to women without HIV *Median [UR]*
**Baseline**	2001 (-20 years)	61.5 [33.1, 101.7]	0.31 [0.24, 0.38]	0.57 [0.41, 0.74]	2.9 [1.8, 4.8]
	2021 (present)	84.2 [58.9, 123.3]	0.35 [0.26, 0.42]	0.73 [0.63, 0.86]	5.6 [3.8, 9.1]
	2031 (+10 years)	69.5 [47.7, 112.4]	0.31 [0.24, 0.39]	0.74 [0.63, 0.85]	6.2 [4.6, 9.5]
	2056 (+25 years)	36.3 [20.6, 67.5]	0.27 [0.21, 0.36]	0.63 [0.54, 0.78]	4.9 [3.6, 6.6]
	2071 (+50 years)	26.9 [13.3, 53.3]	0.25 [0.19, 0.34]	0.58 [0.47, 0.74]	4.3 [3.3, 5.7]
**ART scale-up only**	2021	84.2 [58.9, 123.3]	0.35 [0.26, 0.42]	0.73 [0.63, 0.86]	5.6 [3.8, 9.2]
	2031	69.7 [46.9, 113.7]	0.28 [0.22, 0.35]	0.73 [0.63, 0.84]	6.9 [5.2, 10.5]
	2056	32.5 [15.9, 62.2]	0.19 [0.15, 0.26]	0.58 [0.49, 0.72]	6.1 [4.4, 8.2]
	2071	20.2 [8.0, 45.0]	0.16 [0.11, 0.24]	0.49 [0.38, 0.66]	5.2 [3.7, 7.3]
**Enhanced cervical cancer interventions**	2021	81.9 [57.3, 119.6]	0.35 [0.26, 0.42]	0.73 [0.63, 0.86]	5.6 [3.7, 9.0]
	2031	51.3 [34.8, 80.9]	0.28 [0.22, 0.36]	0.71 [0.62, 0.83]	6.4 [4.9, 9.8]
	2056	10.2 [4.6, 17.4]	0.19 [0.15, 0.26]	0.59 [0.49, 0.74]	6.1 [4.5, 8.4]
	2071	5.3 [2.2, 10.4]	0.16 [0.11, 0.24]	0.48 [0.37, 0.66]	4.8 [3.6, 7.6]
**Enhanced cervical cancer interventions for women living with HIV**	2021	76.5 [53.9, 112.1]	0.35 [0.26, 0.42]	0.72 [0.61, 0.85]	5.1 [3.5, 8.3]
	2031	38.2 [25.5, 57.2]	0.28 [0.22, 0.36]	0.62 [0.52, 0.76]	4.0 [3.0, 6.3]
	2056	5.7 [2.5, 11.8]	0.19 [0.15, 0.27]	0.41 [0.30, 0.59]	3.2 [2.1, 4.0]
	2071	3.4 [1.5, 8.1]	0.17 [0.11, 0.24]	0.32 [0.25, 0.49]	2.7 [1.9, 3.4]

### ART scale-up only scenario

In the ART scale-up only scenario, we projected that the cervical cancer incidence rate among all women will increase by 0.5% [-1.8, 2.1%] from 2021 to 2031, after which it will decline (Fig 1 and Table 2). However, in this scenario, the rate ratio of cervical cancer incidence among women living with HIV relative to women without HIV was predicted to increase and then return by 2071 to a level similar to 2021 (5.2 [3.7, 7.3]). Among women living with HIV, the estimated cervical cancer incidence rate with ART scale-up remained higher than in the baseline scenario for the entire simulated 50-year period. The projected proportion of cervical cancer cases that are women living with HIV declined to 0.73 [0.63, 0.84] by 2031 (within 10 years), 0.58 [0.49, 0.72] by 2056 (within 25 years), and 0.49 [0.38, 0.66] by 2071 (within 50 years) (Fig 2 and Table 2). This decline in the proportion of cases that are women living with HIV coincided with a reduction in HIV prevalence (Fig 2) and an increase in the proportion of women who are virally suppressed of those living with HIV who develop cervical cancer (Fig 11 in S1 Appendix).

### Enhanced cervical cancer interventions scenario

With enhanced cancer prevention interventions for all women, we projected cervical cancer incidence will decline relative to the baseline scenario, both among the total female population and by HIV status. However, disparities in incidence were predicted to persist, with a rate ratio of 4.8 [3.6, 7.6] in 2071 (Fig 1 and Table 2). The proportion of cervical cancer cases that are women living with HIV remained similar to that predicted with ART scale-up only: 0.71 [0.62, 0.83] by 2031 (within 10 years), 0.59 [0.49, 0.74] by 2056 (within 25 years), and 0.48 [0.37, 0.66] by 2071 (within 50 years) (Fig 2 and Table 2).

### Enhanced cervical cancer interventions for women living with HIV scenario

The rate ratio of cervical cancer incidence by HIV status was estimated to decrease to 2.7 [1.9, 3.4] by 2071 in the scenario adding enhanced cervical cancer interventions for women living with HIV (Fig 1 and Table 2). With the addition of catch-up 9vHPV vaccination and more frequent screening for women living with HIV, the proportion of cervical cancer cases that are women living with HIV declined to 0.62 [0.52, 0.76] by 2031 (within 10 years), 0.41 [0.30, 0.59] by 2056 (within 25 years), and 0.32 [0.25, 0.49] by 2071 (within 50 years) (Fig 2 and Table 2).

### Percent change in cervical cancer and HIV outcomes relative to the baseline scenario

**Fig 3:**
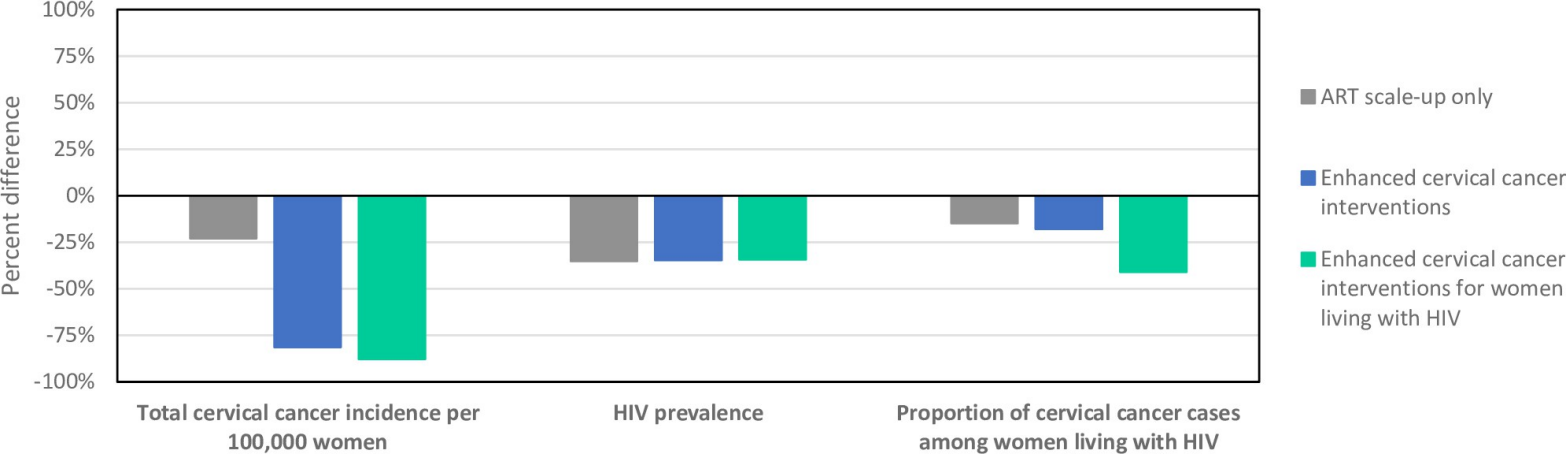
Median percent difference in simulated cervical cancer and HIV outcomes in 2071 with each scenario compared to the baseline scenario in 2071 among women aged 15+.

Relative to the baseline scenario projections for 2071, we estimated that there will be a 23.0% [11.3, 39.9%] decline in total cervical cancer incidence per 100,000 women aged 15+ with ART scale-up only, an 81.4% [73.7, 85.6%] reduction with enhanced cervical cancer interventions, and an 87.7% [77.9, 92.6%] decline with enhanced cervical cancer interventions for women living with HIV (Fig 3). We projected that HIV prevalence among women will decline by a median value of 34.3–35.1% across scenarios, which all include ART scale-up. We predicted that within 50 years, the proportion of cervical cancer cases that are women living with HIV will decline by 14.8% [8.6, 20.4%] with ART scale-up only, 17.9% [7.0, 24.0%] with enhanced cervical cancer interventions, and 41.0% [32.5, 53.0%] with enhanced cervical cancer interventions for women living with HIV.

## Discussion

This modeling analysis provides evidence that disparities in cervical cancer incidence will persist and may even increase with ART-scale-up. Scaling up HPV vaccination and cervical cancer screening tailored by HIV status will decrease total cancer incidence and reduce but not remove the disproportionate cervical cancer burden among women living with HIV. In 2021, we estimated that the 35% of women aged 15+ living with HIV account for 73% of cervical cancer cases. Compared to the status quo scenario, the 2071 rate ratio of cervical cancer incidence among women living HIV relative to those without can be decreased by approximately 40% by increasing preventative interventions for women living with HIV. Under this enhanced scenario, we predicted that the 17% of women aged 15+ will be living with HIV and will account for 32% of cervical cancer cases by 2071.

Our modeled estimates of the disproportionate burden of cervical cancer incidence in women living with HIV are consistent with previously published values. In 2021, we predicted that 73% [63%, 86%] of new cervical cancer cases in KwaZulu-Natal will be women living with HIV, and that women living with HIV experience a cervical cancer incidence rate 5.6 [3.8, 9.1] times higher than women without HIV. By comparison, a recent systematic review and meta-analysis of the association between HIV infection and cervical cancer estimated that in 2018, 63.4% (95% CI: 55.2–70.7%) of new cervical cancer cases in South Africa were women living with HIV [2]. We expect a greater proportion of cases among women living with HIV in KwaZulu-Natal than in South Africa due to higher HIV prevalence (37.3% of reproductive-aged women were living with HIV in KwaZulu-Natal, compared to 27.3% nationally, in 2016) [16]. The meta-analysis also estimated that, on average, women living with HIV in low- or middle-income countries (LMICs) have 6.79 (95% CI: 3.89–11.84) times the cervical cancer risk of women without HIV [2]. This 95% CI contains our estimated incidence rate ratio of 5.6 [3.8, 9.1].

Our results illustrate the complex effects of ART on cervical cancer incidence. In the ART scale-up only scenario, we projected that from 2021 to 2071, the cervical cancer incidence rate among women living with HIV will remain higher than the corresponding rate in the baseline scenario. This occurs because viral suppression reduces HIV-associated mortality as a competing risk and more women living with HIV survive to develop cervical cancer. In contrast to the heightened cervical cancer incidence among women living with HIV, we predicted that a scenario with scale-up of ART only will reduce cervical cancer incidence among the total female population. Scale-up of ART and subsequent viral suppression decrease both HIV incidence and the number of women living with untreated HIV, who are at high risk of cervical cancer. The countervailing effects of ART have been demonstrated using other models of cervical cancer incidence in South Africa [6] and Tanzania [7]. Similar to our model, these simulations show that ART scale-up is associated with a short-term increase in cervical cancer incidence but contributes to reducing population-level cancer burden in the long term.

In 2018, the World Health Organization (WHO) Director-General issued a global call for cervical cancer elimination and established guidelines for reaching this goal [22]. The WHO intervention package recommends 90% HPV vaccination coverage of girls by age 15, 70% cervical cancer screening coverage with a high-performance test by ages 35 and 45, and 90% treatment coverage for women with precancer or management of invasive cancers by 2030 [23]. Other models have shown that adoption of the WHO intervention package achieves cervical cancer elimination in countries with a high burden of cervical cancer by the end of the century [24], and that further increasing vaccination or screening frequency for women living with HIV can significantly decrease near-term cervical cancer incidence [6, 13–15]. The HPV vaccination and cervical cancer screening components of our enhanced cervical cancer interventions scenario map to the WHO recommendations, although we model more conservative levels of precancer and cervical cancer treatment (95% of eligible women receive thermal ablation, 80% of those ineligible for ablation receive LLETZ, and 40% of women diagnosed with cervical cancer receive a hysterectomy), and do not assume 100% treatment efficacy for precancer as in previous modeling. Our analysis advances previous work by underscoring the role of catch-up vaccination and more frequent screening in reducing the elevated burden of cervical cancer incidence among women living with HIV. Although current practice of cytology with colposcopy triage has not facilitated fidelity to guidelines, particularly for women living with HIV [19], a switch to a single-visit strategy may make scale-up of repeat screening more feasible.

Integrating cervical cancer prevention and treatment with HIV care may increase receipt of both types of services by women living with HIV. Under the baseline scenario, we projected that the proportion of cervical cancer cases in women living with HIV that are women with HIV viral suppression ranges from 0.66 [0.61, 0.69] in 2021 to 0.62 [0.54, 0.70] in 2071. In the alternative scenarios, this proportion consistently increased to median values of 0.74–0.76 in 2031, and to 0.66–0.77 in 2071. These results suggest that incorporating cervical cancer interventions into existing HIV care systems can potentially reach a large proportion of women living with HIV. Co-located HIV treatment and cervical cancer prevention clinics have been successfully implemented in Zambia [25–27]. In a qualitative study of perceptions of integrated cervical cancer and HIV screening in Uganda, mentioned benefits included reduced time, transportation costs, and stress, and increased screening coverage and confidentiality for the women screened [28]. Despite these potential strengths, focusing enhanced cervical cancer prevention and treatment programs toward women engaged in HIV care will miss a significant group of women living with unsuppressed HIV who are at high risk for cervical cancer. Different strategies may be needed to identify and link women without consistent HIV care to cervical cancer prevention and treatment. Programs may also consider a reverse approach that offers HIV treatment within less-stigmatized cervical cancer services. Critically, scale-up of ART and adherence support must be foundational to any program providing services to women living with HIV. Future work can use cost-effectiveness analyses and implementation science to identify service delivery approaches that effectively use limited health system resources to increase uptake and retention in both HIV and cervical cancer services.

A strength of this study is that the model was specifically developed to account for HIV-HPV coinfection dynamics and calibrated using a robust Bayesian approach. Additionally, model parameters accounted for differential cervical cancer intervention effectiveness by HIV status, allowing us to compare the impact of intervention scenarios on the disproportionate burden of cervical cancer in women living with HIV. By presenting outcomes stratified by HIV and ART status, our results can be used to inform resource allocation and clinical service strategies.

There are several limitations to the modeled estimates. First, there is considerable uncertainty in model parameters for sexual behavior and natural history of HIV and HPV. However, by using a multiple-parameter Bayesian calibration procedure and presenting uncertainty ranges from simulations using a range of parameter combinations, our results provide a more complete picture of potential cervical cancer trends. In addition to historic and current parameter values, sexual behavior and the underlying landscape of HIV and cervical cancer prevention and treatment may change over the next 50 years. Uncertainty in future parameters increases with time, and we present intermediate projections alongside 50-year outcomes. Second, due to our compartmental model structure, we were unable to simulate differences in screening behavior based on individual screening history. Future work to explore the impact of individual screening trajectories on population-level cervical cancer incidence will be valuable. Third, our scenarios include scale-up of ART as treatment through policies such as universal-test-and-treat, but we do not model use of oral and injectable HIV pre-exposure prophylaxis (PrEP). A recent study conducted in Uganda and Kenya estimated a 76% decrease in HIV incidence among women initiating PrEP compared to matched controls [29]. Scale-up of PrEP was prioritized in South Africa’s 2017–2022 strategic plan to prevent new HIV infections [30], and our predictions may overestimate the proportion of cervical cancer cases that are women with HIV if PrEP further decreases HIV incidence. However, cervical cancer is a slow-progressing disease and our intermediate-term predictions will be less influenced by PrEP scale-up. Fourth, there is uncertainty about the effectiveness of catch-up vaccination among women living with HIV [31, 32]. If effectiveness is lower among this group, our projections may overestimate the impact of enhanced cervical cancer interventions for women living with HIV. Finally, our scenarios assume that South Africa switches from using the 2v to the 9vHPV vaccine. If South Africa does not make this change, our estimates may overestimate vaccine impact, although the 2v vaccine provides substantial cross-protection against additional hrHPV types included in the 9v vaccine [33]. Additionally, growing evidence suggests that a single dose of either the 2v or 9v vaccine is highly effective in preventing persistent HPV infection, similar to multidose regimens [34]. A future switch to a single-dose HPV vaccine regimen could increase vaccine coverage and decrease the overall burden of HPV.

## Conclusions

Overall, our results suggest that, in the context of scaled-up HIV interventions, enhanced cervical cancer prevention for women living with HIV will be necessary to alleviate disparities in cervical cancer incidence by HIV status. Although there has been growing investment in reducing cancer incidence worldwide, cervical cancer burden is increasingly concentrated among vulnerable subgroups of people. To reduce total cervical cancer incidence in settings with high HIV prevalence, enhanced services will be needed to decrease cancer incidence among women at high risk for cancer. But even cervical cancer services tailored for women living with HIV may not eliminate disparities: continued efforts are needed to fill gaps in prevention and treatment of cervical cancer and HIV in Southern Africa.

## Supporting information

S1 Appendix (PDF)
