# Supplementary material for: Enhanced cervical cancer and HIV interventions reduce the disproportionate burden of cervical cancer cases among women living with HIV: A modeling analysis
Source: PLoS One. 2024 May 23;19(5):e0301997. doi: 10.1371/journal.pone.0301997 (PMC11115290; doi:10.1371/journal.pone.0301997)
Supplement: S1 Appendix — (PDF) [file pone.0301997.s001.pdf]

Supplemental Appendix

Supplement to: Broshkevitch CJ, Barnabas RV, Liu G, Palanee-Phillips T, Rao DW. Enhanced cervical cancer and HIV interventions reduce the disproportionate burden of cervical cancer cases among women living with HIV: A modeling analysis

Table of Contents

I. Model overview..........3

II. Future interventions to prevent and treat HIV and HPV/cervical cancer..........3

II.a. Future scenarios for HIV antiretroviral therapy (ART)..........3

II.b. Future scenarios for cervical cancer screening, treatment, and HPV vaccination..........3

III. Calibration and Validation..........7

IV. Additional Model Output..........15

V. References..........16

I. Model overview

The *DRIVE (Data-driven Recommendations for Interventions against Viral infEction)* model is a deterministic, compartmental model parameterized to represent transmission of human papillomavirus (HPV) and progression to cervical cancer in KwaZulu-Natal, South Africa (KZN), a region with high HIV prevalence.[1,2] The model was originally described in 2018,[3] but an adapted model was published in 2022 alongside a comprehensive technical appendix.[4] Section II of this appendix details the future HIV antiretroviral therapy (ART), HPV vaccination, and cervical cancer screening and treatment interventions analyzed in the present paper, which differ from those examined previously. Section III shows model fit to select calibration and validation data on HIV prevalence and cervical cancer incidence. Some text for this model overview and the cervical cancer screening and treatment section, as well as the Section III figures, are repeated from the 2022 published technical appendix.[4]

The primary objectives of the model are to evaluate and predict the impact of HPV vaccination, cervical cancer screening and treatment, and HIV prevention and treatment on cervical cancer outcomes. Beginning in 1925, the model generates a population stratified by age, gender and sexual risk group. This population engages in heterosexual transmission of oncogenic HPV (high-risk HPV; hrHPV) and, beginning in 1980, HIV infection.

HIV progression is tracked by both CD4+ T-cell (CD4) count and HIV RNA concentration (viral load), and infected individuals may achieve viral suppression with initiation of ART, beginning in 2004. Men and women infected with hrHPV may clear the infection, and infections in women may progress through stages of precancerous lesions to cervical cancer. A key feature of our model is representation of the interaction between HIV and HPV, whereby HIV infection increases the risk of HPV acquisition and the rate of disease progression. The model is calibrated to reproduce observed population-level HPV and HIV dynamics using maximum-likelihood estimation.

Model dynamics are governed by a system of differential equations that are solved in MATLAB using a 4th-order Runge-Kutta numerical method. HPV is introduced in 1925 to allow HPV transmission dynamics and cervical cancer incidence to equilibrate prior to the introduction of HIV infection in 1980. The model simulates events in discrete time with two-month intervals. At each time step, differential equations are evaluated to estimate population demographics and the number of persons in each infection, disease, or treatment state for the following time step. The dynamic nature of our transmission model captures population-level effects such as herd immunity.

This work was facilitated through the use of advanced computational, storage, and networking infrastructure provided by the Hyak supercomputer system at the University of Washington.

II. Future interventions to prevent and treat HIV and HPV/cervical cancer

II.a. Future scenarios for HIV antiretroviral therapy (ART)

In our baseline simulation projecting forward in time, we assume no future scale-up of ART. The proportion of persons living with HIV who are virally suppressed remains at the estimated levels for 2017 for the duration of the simulation. In our enhanced intervention scenarios, we assume ART is scaled up to the UNAIDS 90-90-90 targets (72.9% of persons with HIV virally suppressed) between 2021 and 2030.

II.b. Future scenarios for cervical cancer screening, treatment, and HPV vaccination

We model four primary scenarios with differing coverage and loss to follow-up for screening and treatment, and coverage of HPV vaccination. The baseline scenario and ART scale-up only scenarios are a continuation of historical practice: cervical screening is with cytology, individuals with AS-CUS+ are referred for colposcopic evaluation with biopsy, and those found to have high-grade lesions are treated with LLETZ or cryotherapy. Although guidelines call for repeat screening,[5] we make a conservative assumption of once-lifetime screening between ages 35-39 at 48% coverage[6,7] as observed data suggest low compliance with the recommended schedule.[8,91,21,2] In this scenario, 57% of girls aged 9-14 are vaccinated,[10] although we assume a switch from the bivalent to the nonavalent vaccine in 2021. If this change does not occur in reality, we would expect cervical cancer incidence to decline to a lesser extent than projected. However, we also assume no protection with a single dose of the vaccine; as of 2018, an estimated 71% of girls in South Africa had received at least one dose, and there is evidence that single-dose vaccination may provide substantial protection.[11,12] We assume the vaccine is 100% effective against covered types (HPV16/18/31/33/54/52/58) for girls who are uninfected with those types at the time of vaccination, and the duration of protection is lifelong.

In our enhanced cervical cancer intervention scenarios, effective vaccine coverage is scaled up to 90% for girls aged 9-14 beginning in 2021, again with the nonavalent vaccine. If evidence more conclusively demonstrates the efficacy of single-dose vaccination, current levels of engagement would provide a solid foundation for reaching this benchmark. In the enhanced cervical cancer interventions for all women scenario, we additionally model implementation of a single-visit strategy for cervical cancer screening and treatment: twice-per-lifetime primary HPV DNA testing at ages 35-39 and 45-49, with immediate treatment of individuals who screen positive for high-risk HPV. In the enhanced cervical cancer interventions for women with HIV scenario, women with HIV aged 15-24 receive additional catch-up vaccination with 50% coverage. Women without HIV continue to receive HPV DNA testing twice-per-lifetime, while women with HIV are tested every five years. In both of these scenarios, the proportion of women screened in each age group is scaled up from 48% in 2021, to 70% in 2030, and to 90% in 2045.

Although women with HIV are recommended to screen every 3 years,[5] given the structure of our model with 5-year age groupings, we approximate this by implementing screening in each 5-year group. Additionally, because our model is compartmental and doesn't track screening history, a random subset of individuals is screened in each age group. This implies that any given individual may not screen at each 5- or 10-year interval. In all scenarios, we assume that ART-naïve women living with HIV are equally likely to screen as treated women living with HIV. In reality, untreated women may be more difficult to engage in repeat screening,[13] which would lessen the impact of the repeat screening strategies.

Based on a review of the literature, we defined model inputs governing the sensitivity and specificity of screening and diagnostic tests and the effectiveness of treatment to vary by HIV status. Table 1 shows the parameters that distinguish the interventions used in our scenarios. For HPV-based testing, specificity was derived from model results. As discussed in more detail below, because our model simulates and tracks HPV infection and progression through CIN states, we controlled test performance by specifying the proportion of individuals with any high-risk HPV infection. Specificity at each time point is then derived based on the prevalence of (type-specific) HPV infection and the proportion assumed to screen positive.

Differences in screening test performance by HIV and ART status:

There is mixed evidence on whether the performance of cytology depends on HIV status. Several studies have shown that overall agreement between cytology and histology is low for women living with HIV.[14,15] Differences in sensitivity by HIV status in many of these studies have not been significant, but sample sizes have been small.[15-17] There is suggestion of lower specificity among women living with HIV,[15,16] and studies have also shown lower specificity with decreasing CD4 count.[18] We assume slightly lower sensitivity and specificity in women living with HIV to capture these potential differences, as a key focus of this analysis is understanding the impact of cervical cancer prevention strategies in the context of high HIV prevalence. For diagnostic accuracy among HIV-uninfected women, we use the values reported by Arbyn et al.,[19] as the included studies were in settings where HIV prevalence is low. We derive estimates for women living with HIV by applying the relative sensitivity[15,16] and specificity[15] observed by HIV status in available studies.

In the scenarios with cytology-based screening, we modeled triage with colposcopy. The South African guidelines recommend colposcopy with biopsy, but acknowledge that this may not always be feasible due to limited availability of equipment and laboratory care.[20] However, for this analysis, we will conservatively model the guidelines. We assume that colposcopy-guided biopsy has 100% sensitivity and specificity for all women.

HPV DNA testing consistently demonstrates lower specificity for women living with HIV, and there is a suggestion of higher sensitivity.[21-23] There is also evidence that the specificity decreases with lower CD4 count among women living with HIV.[18,24,25] We used estimates of sensitivity by HIV status from a meta-analysis by Kelly et al., [21] which align with estimates from other studies and reviews.[22,26] These values determine the proportion of women in CIN2+ states who screen positive. We additionally made the simplifying assumption that these estimates of sensitivity for CIN2+ correspond to the proportion of individuals with HPV who test positive across disease states. We assume that no individuals without an active HPV infection test positive. We then derived the specificity for HPV DNA testing by counting the number of women in states $\leq$CIN1 who would screen negative divided by the total number of women in states $\leq$CIN1 who were screened.

We assumed that estimates presented for women living with HIV correspond to women with detectable viral load. On the basis of evidence that test performance for women with HIV who have higher CD4 count trends in the direction of performance for HIV-uninfected women,[18,25] we assumed that test performance values for women with viral suppression fall between the estimates for women without HIV and women with untreated HIV.

Treatment retention and effectiveness

In the enhanced cervical cancer intervention scenarios, treatment is with thermal ablation as an alternative to cryotherapy, or with LLETZ for women with lesions ineligible for ablative therapy. Studies have reported comparable effectiveness with cryotherapy and thermal ablation,[27,28] but thermal ablation is more efficient and more scalable – it can

be implemented using portable devices and does not require refrigerant gas, the need for which has limited access to treatment in some settings.[29,30] We therefore assume that use of thermal ablation would facilitate higher retention for treatment following positive screening tests in these scenarios. Assumptions about retention in these scenarios are informed by studies reporting on the percent of women lost to follow-up in screen-and-treat programs. In several, these data showed retention of ~90% or higher,[31-33] although others have reported retention of 43-66% of those offered cryotherapy.[29,30,34] Those reporting lower retention highlight issues in service delivery specific to cryotherapy, suggesting that higher retention may be feasible with thermal ablation.[29,30] Retention appears to be lower for LLETZ than cryotherapy, which is as expected given that LLETZ usually requires a referral.[30,33]

For HPV-based testing, we assume that a visual examination (i.e., visual inspection with acetic acid) will be performed to determine eligibility for ablative therapy. Studies have reported the percentage of women requiring excisional treatment (LLETZ) in the range of 12%-35%[28,31,34-38] without stratification by disease stage. Although there is evidence that women living with HIV are more likely to have larger lesions ineligible for ablative therapy,[35] it is not clear to what extent this reflects women living with HIV having more progressed disease at the time of screening. We thus assume that, within a CIN state, the proportion of women requiring LLETZ is the same across HIV status groups. We assume that 0% of women in CIN1, 10% of women in CIN2, and 30% of women in CIN3 require LLETZ.

In a meta-analysis by Debeaudrap et al.,[39] treatment efficacy was similar for cryotherapy and LLETZ among women living with HIV. There were marginally significant differences when restricted to women treated for high-grade lesions, however, with more treatment failure following cryotherapy. Treatment failure was also higher, but non-significantly so, for treatment of high-grade compared to low-grade lesions. Overall, women living with HIV were twice as likely to experience treatment failure as HIV uninfected women, and this association was not statistically significantly different by treatment method. The authors didn't have enough data to examine differences by ART status or CD4, but some studies included the review reported higher treatment failure with lower CD4 count. Studies comparing thermal ablation with cryotherapy have reported that these methods have similar efficacy as well, both overall and among women living with HIV.[27,28] For simplicity, we assumed the same treatment efficacy for all treatment methods and for all CIN states. We further assumed that estimates for women with HIV apply to women with untreated HIV, and treatment efficacy for women with viral suppression falls between values for women without HIV and those with untreated HIV.

In addition to treatment failure (resulting in women remaining in the CIN compartment in which they were treated), we accounted for persistent HPV post-treatment among those who clear their lesion. From a 2017 systematic review by Hoffman and colleagues, the median HPV persistence at 3 months post-treatment (including women with residual or recurrent CIN) is 48% for cryotherapy and 28% for LLETZ.[40] It is worth noting that in this review, the cryotherapy estimate was based on data from only 1 study, and estimates of type-specific persistence are not presented by treatment method (overall, type-specific persistence was considerably lower than overall persistence at 18% vs. 28-30%). In a study in Zambia, the proportion of participants with persistent hrHPV post-treatment was similar for cryotherapy, thermal ablation, and LLETZ (60%, 58%, and 53%; p=0.48).[28] However, the sample in this study was 50% HIV-positive and measures were taken at 6 months post-treatment, so the higher estimates may reflect re-infection as well as persistence. Despite the limitations, we used the estimates from Hoffman et al.[40] and assumed that persistence is the same with thermal ablation as cryotherapy. The data in this review were from studies in primarily HIV-negative or unknown status samples, however. Applying the same values for all women would mean that fewer women living with HIV have persistent HPV after successful treatment, since the percent of women who have persistent HPV but are cleared of CIN is equal to the overall percent persistent minus (1- treatment efficacy), and treatment efficacy is lower for women living with HIV. Lacking data on persistence stratified by HIV status, we used the data from this review to estimate the proportion of treated HIV-negative women who would have persistent HPV without CIN and applied that same percentage to women with HIV.

Table 1: Key parameters for modeled scenarios, by groups defined by HIV status and viral suppression from antiretroviral therapy

	**Women without HIV**	**Untreated women with HIV**	**Virally suppressed women with HIV[a]**	**Refs**
**Screening and triage performance characteristics[b]**				
Cytology				
Sensitivity[c]	0.57	0.52	0.55	15,16,19
Specificity[c]	0.93	0.85	0.89	3–515,16,19
Colposcopy				
Sensitivity	1.0	1.0	1.0	Asm
Specificity	1.0	1.0	1.0	Asm
HPV DNA testing				
Sensitivity	0.85	0.94	0.90	21
Specificity [d]	derived	derived	derived	Derived
**Treatment**				
Treatment failure (all methods)[e]	9%	23%	16%	39
HPV persistence (ablative therapies)[f]	39%	39%	39%	40
HPV persistence (LLETZ)[f]	19%	19%	19%	40
**Loss to follow-up**				
Three-visit strategy				
Colposcopy	28%	28%	28%	41
Treatment	50%	50%	50%	41
Single-visit strategies				
Thermal ablation	5%	5%	5%	Asm
LLETZ[g]	20%	20%	20%	Asm

Acronyms and abbreviations: Asm, assumption; HPV, human papillomavirus; LLETZ, large loop excision of the transformation zone; Refs, references

[a]Test performance and treatment efficacy for women with viral suppression is assumed to fall between values for HIV-negative and untreated HIV-positive populations; [b]Sensitivity and specificity are defined with reference to CIN2+ as the disease state; [c]Threshold of ASCUS+. [d]Test performance is derived from the model and varies over time with changes in HPV prevalence and type distribution as HPV vaccination is scaled up. [e]Treatment failure refers to the proportion treated with CIN who remain in that CIN state post-treatment. [f]HPV persistence refers to the proportion of women treated whose lesions resolve but who remain HPV-positive; [g]Based on evidence from screen-and-treat programs reporting high retention in treatment,[31-33] we assume that 95% of screen-positive women with eligible lesions receive treatment with thermal ablation. Receipt of treatment is lower among women requiring referral to LLETZ.[30,33,42]

III. Calibration and Validation

Model fit to select calibration and validation data on HIV prevalence and cervical cancer incidence. For complete details on model calibration and validation, as well as model fit to observed HPV and CIN data, see the comprehensive model technical appendix published in 2022.[4]

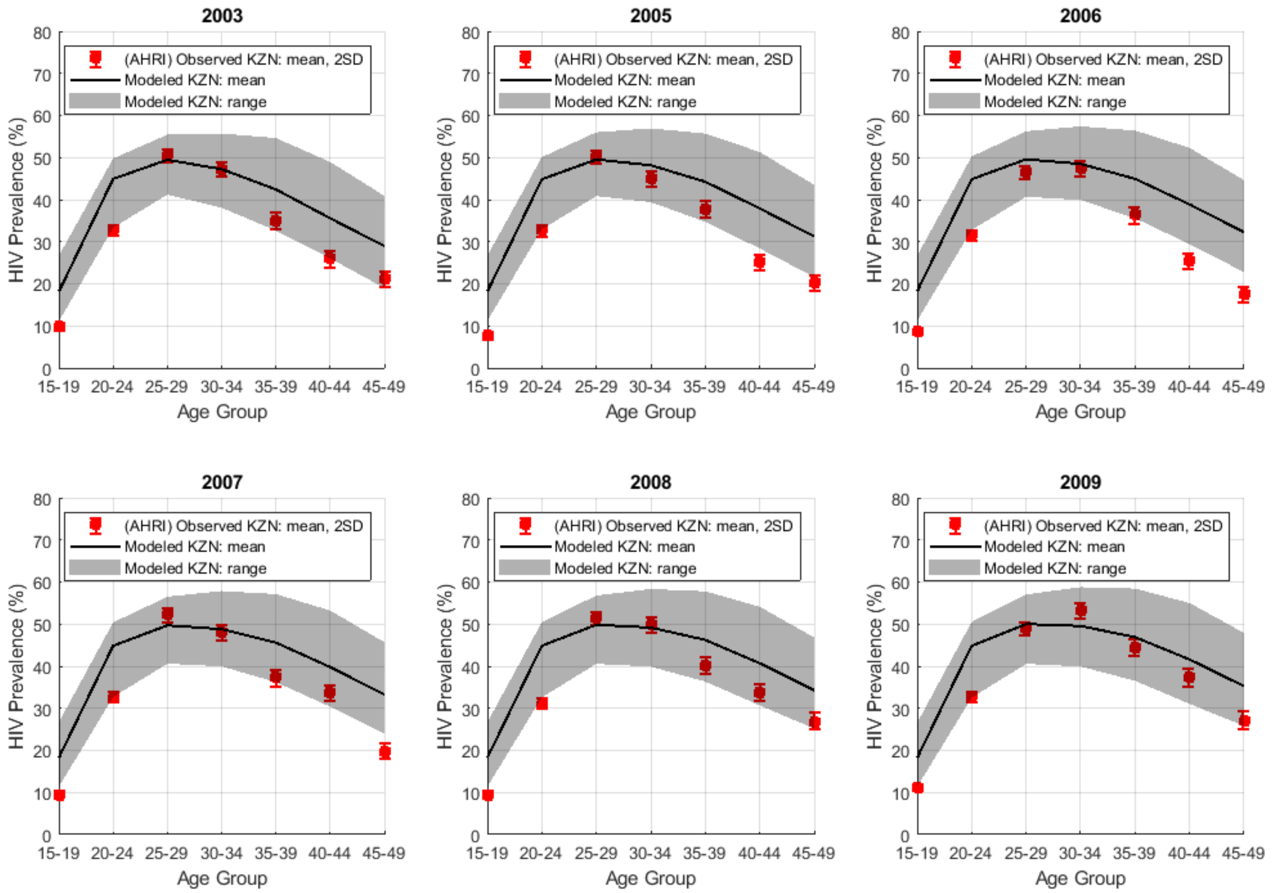

Figure 1. Model fit to observed HIV prevalence data in women by age over time. Standard deviation of the observed data calculated assuming that prevalence proportions follow a normal approximation of the binomial distribution. Shaded regions represent the range of estimates using the 25 best-fitting model parameter sets.

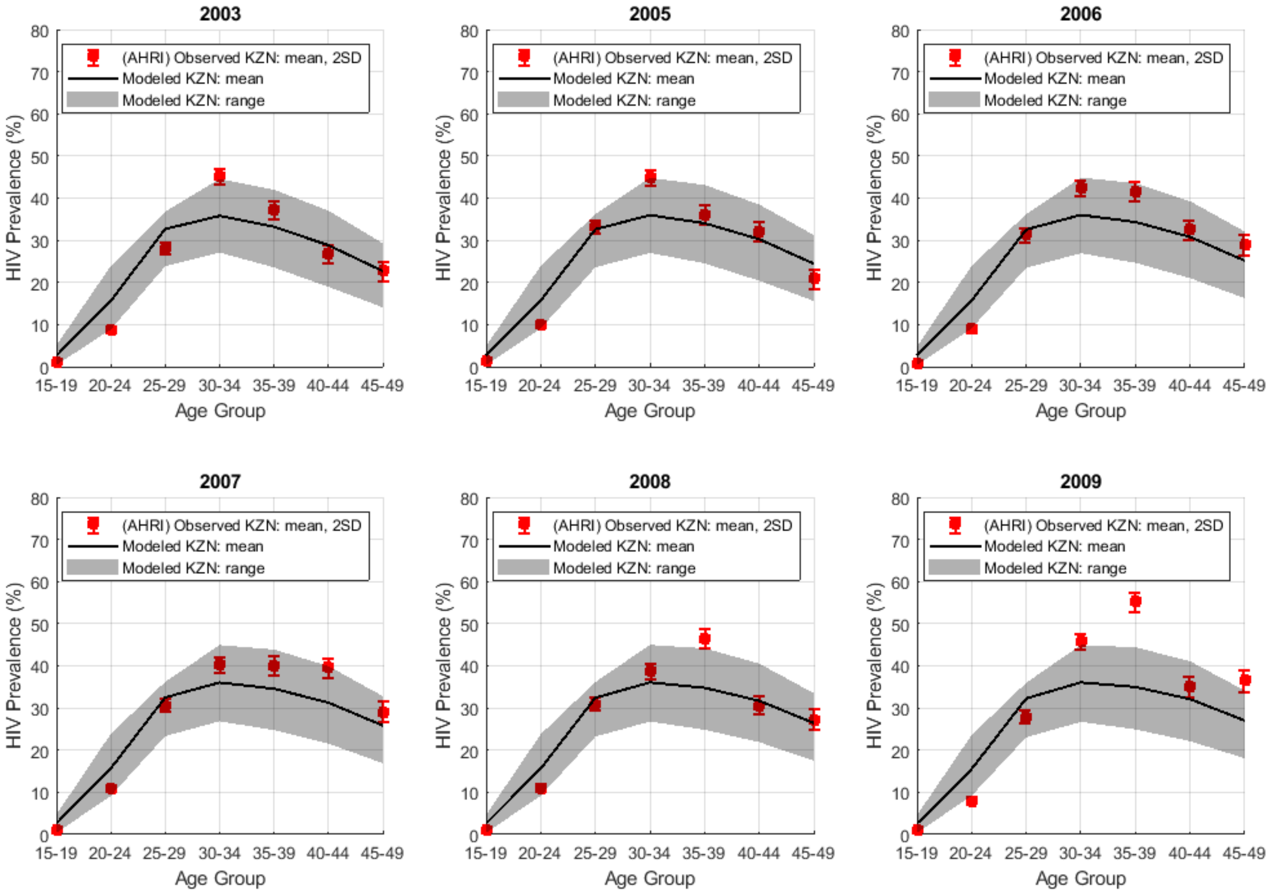

Figure 2. Model fit to observed HIV prevalence data in men by age over time. Standard deviation of the observed data calculated assuming that prevalence proportions follow a normal approximation of the binomial distribution. Shaded regions represent the range of estimates using the 25 best-fitting model parameter sets.

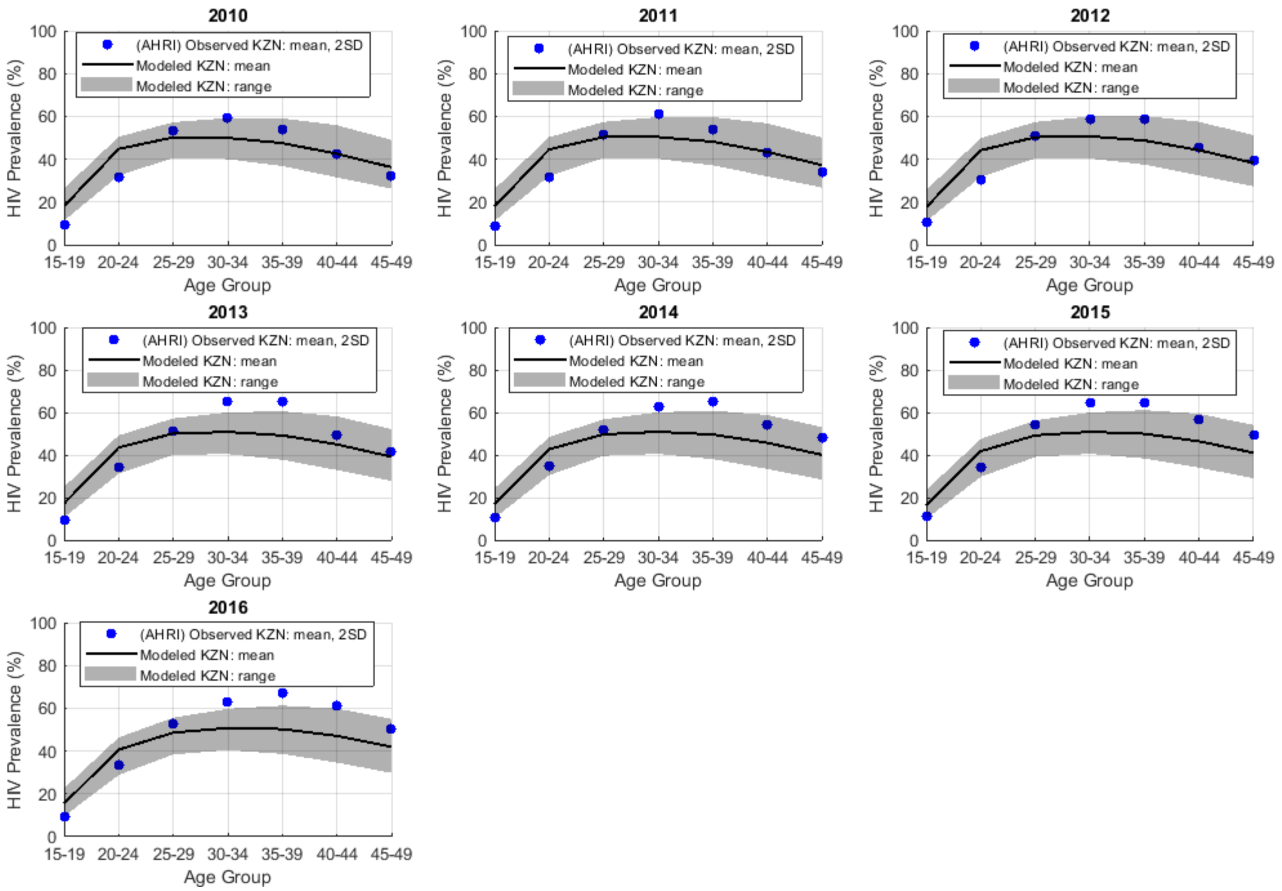

Figure 3. Model validation to observed HIV prevalence data in women by age over time. Shaded regions represent the range of estimates using the 25 best-fitting model parameter sets.

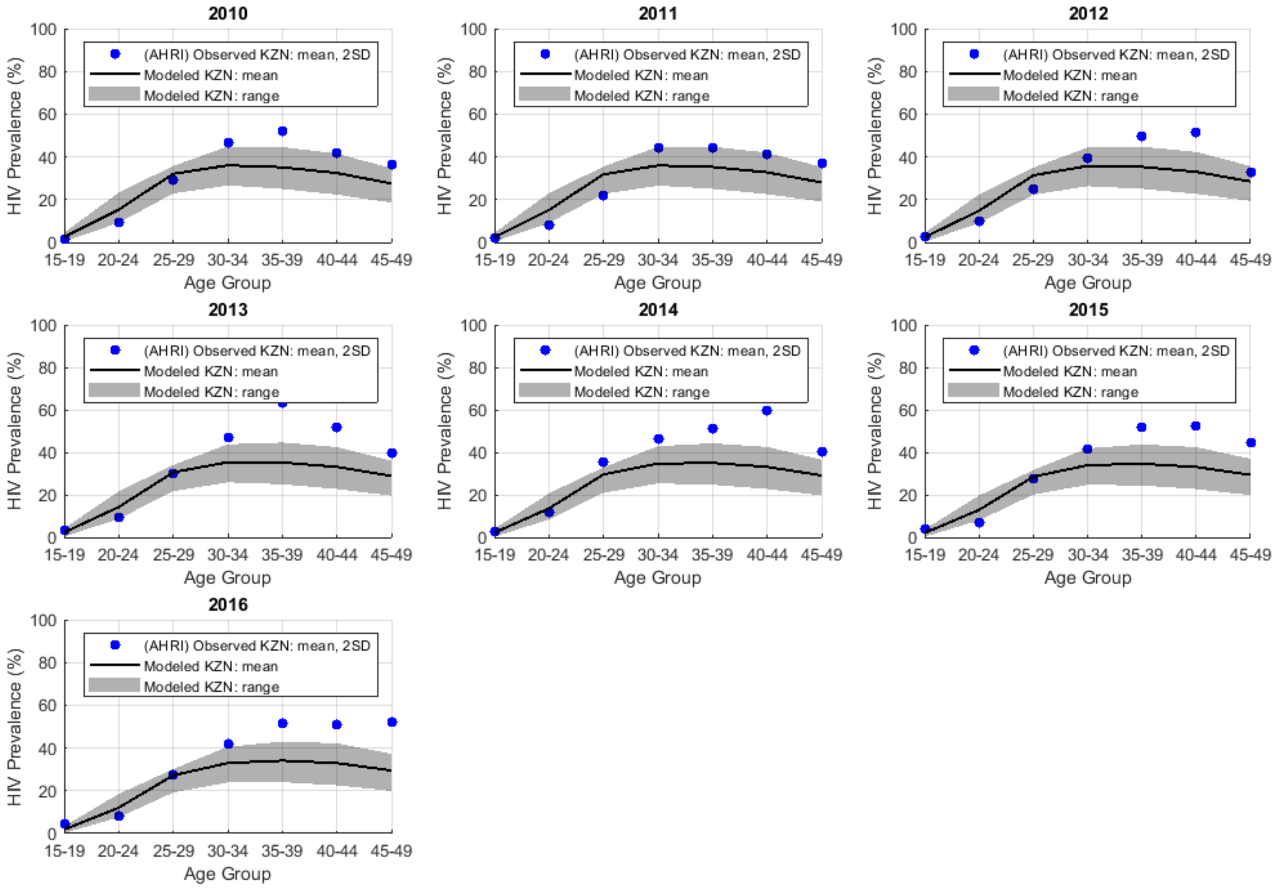

Figure 4. Model validation to observed HIV prevalence data in men by age over time. Shaded regions represent the range of estimates using the 25 best-fitting model parameter sets.

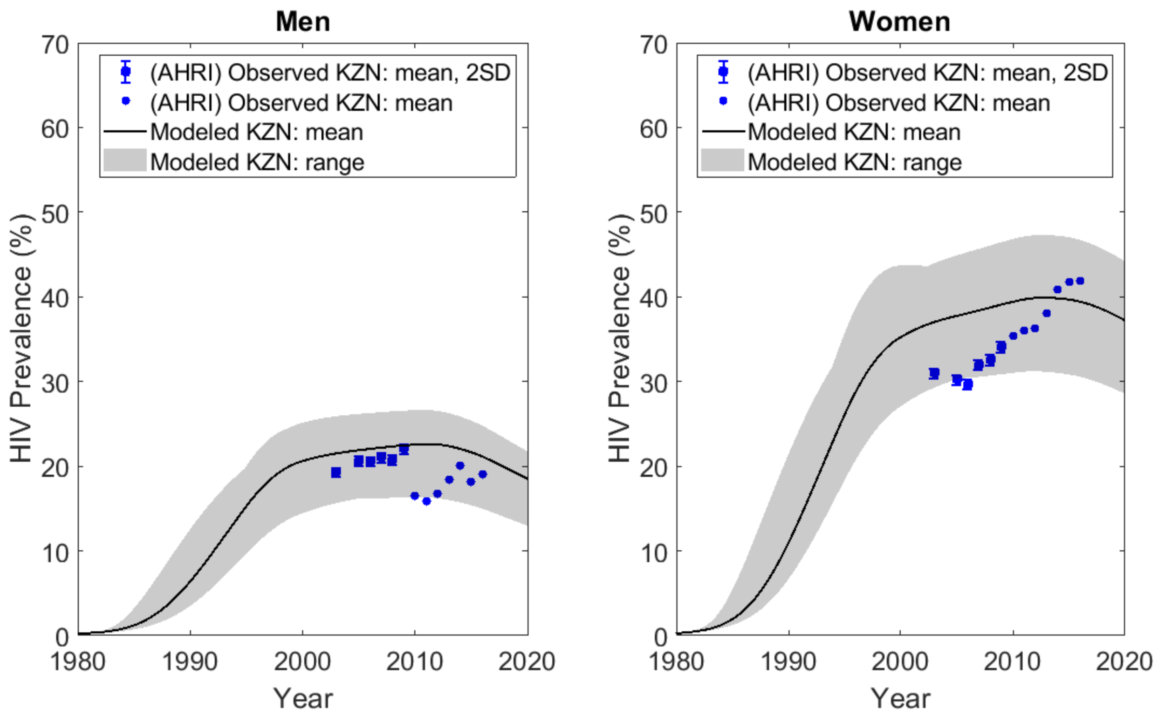

Figure 5. Model validation to observed HIV prevalence data by gender over time. Blue error bars from 2003-2009 recalculate HIV prevalence for ages 15-49 combined from the calibration dataset, while blue data from 2010-2016 represents later timepoints used only for validation. Shaded regions represent the range of estimates using the 25 best-fitting model parameter sets.

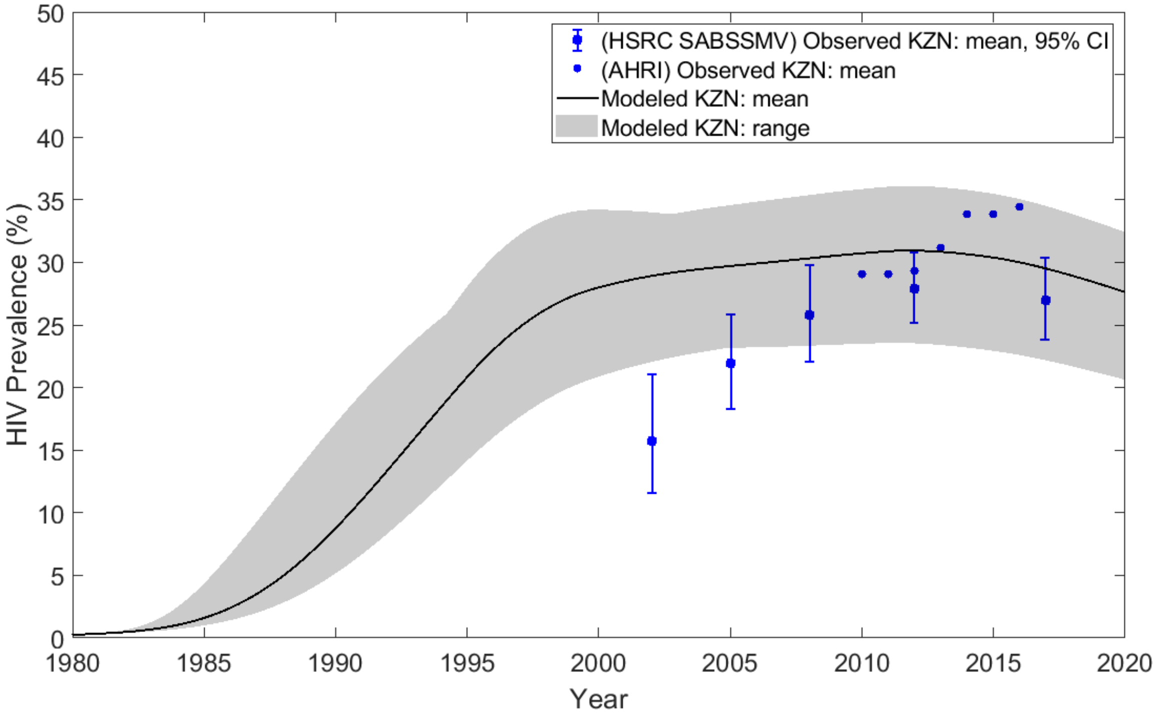

Figure 6. Model validation to observed HIV prevalence data among ages 15-49 over time. Shaded region represents the range of estimates using the 25 best-fitting model parameter sets.

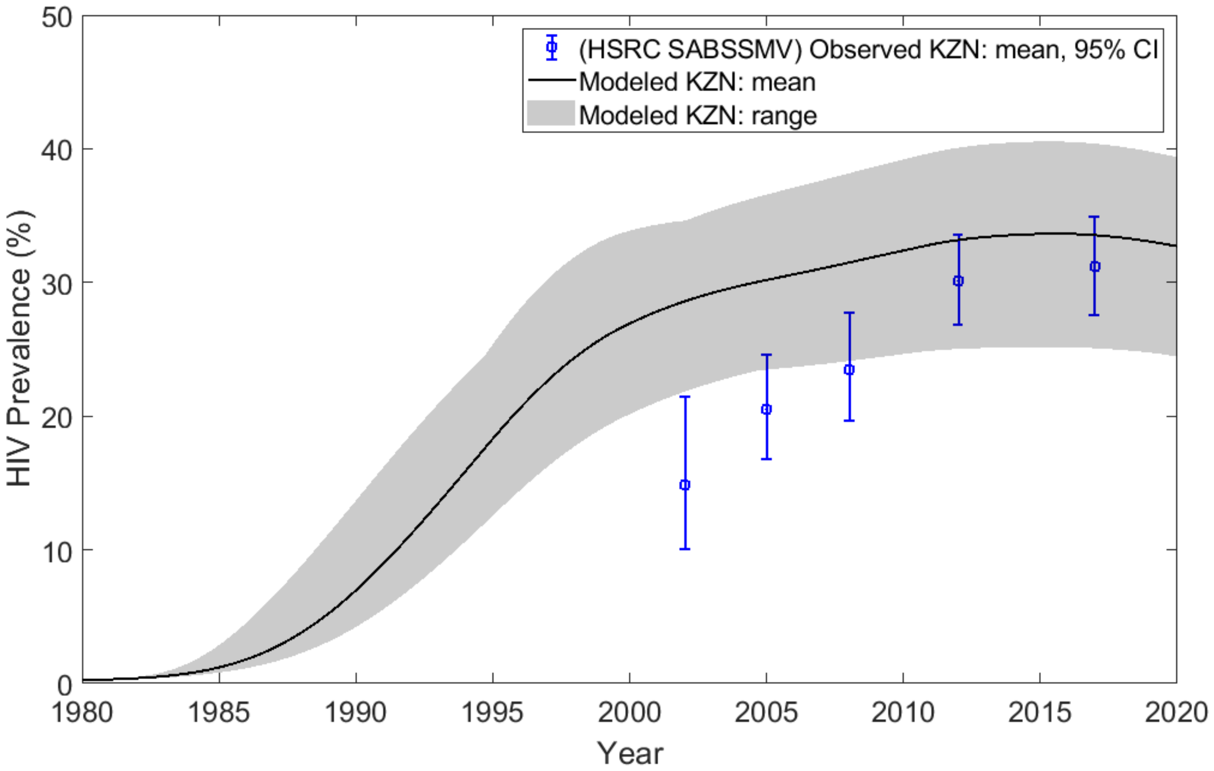

Figure 7. Model validation to observed HIV prevalence data among ages 25+ over time. Shaded region represents the range of estimates using the 25 best-fitting model parameter sets.

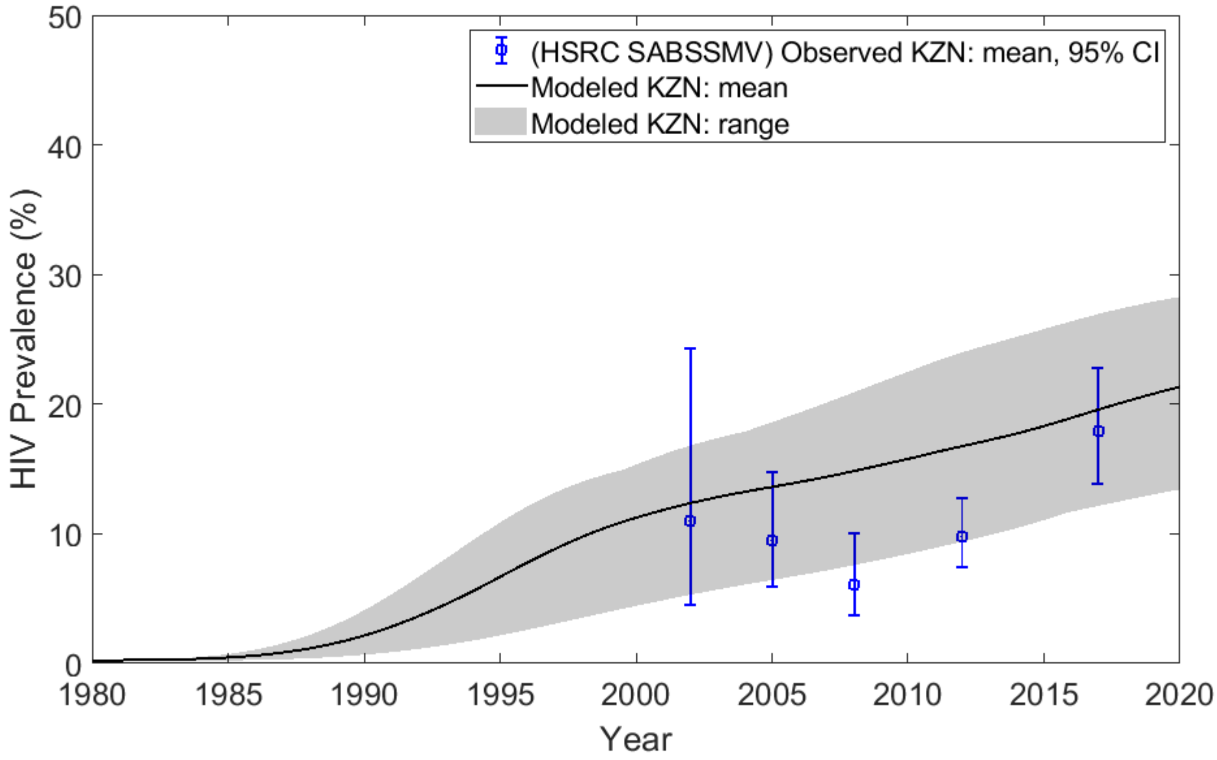

Figure 8. Model validation to observed HIV prevalence data among ages 50+ over time. Shaded region represents the range of estimates using the 25 best-fitting model parameter sets.

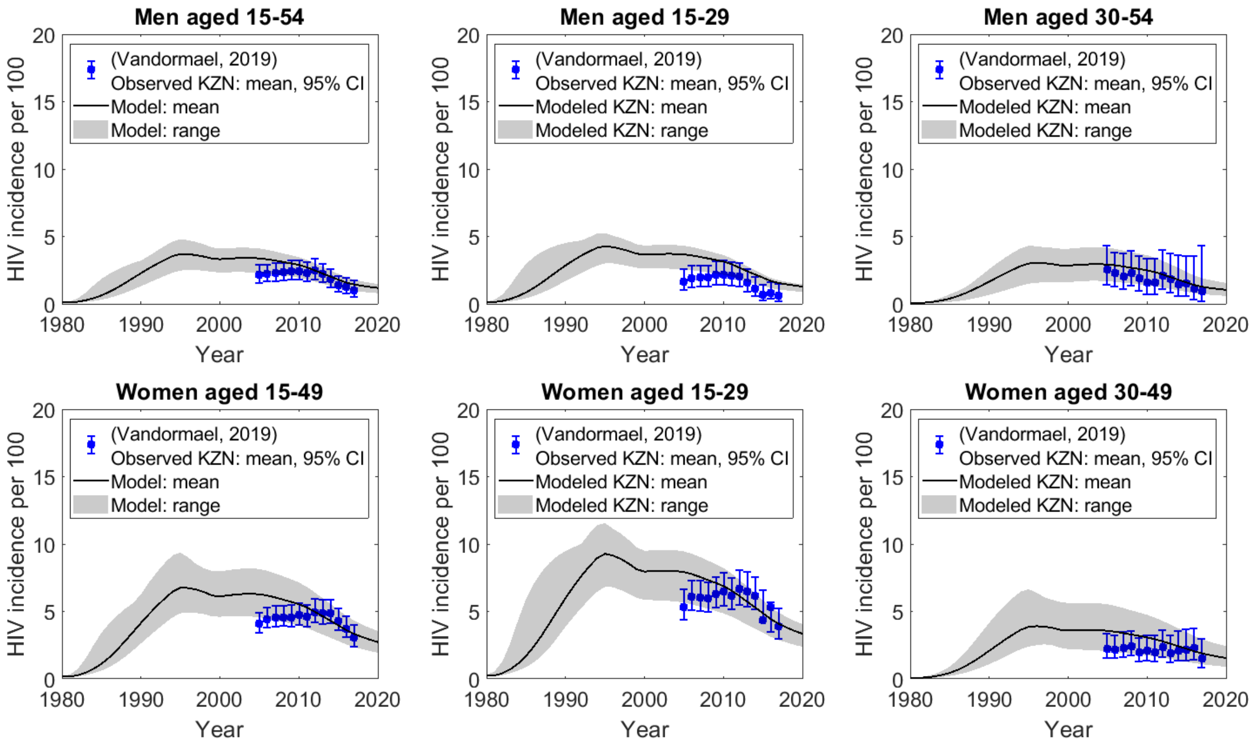

Figure 9. Model validation to observed HIV incidence data by gender and age over time. Shaded regions represent the range of estimates using the 25 best-fitting model parameter sets.

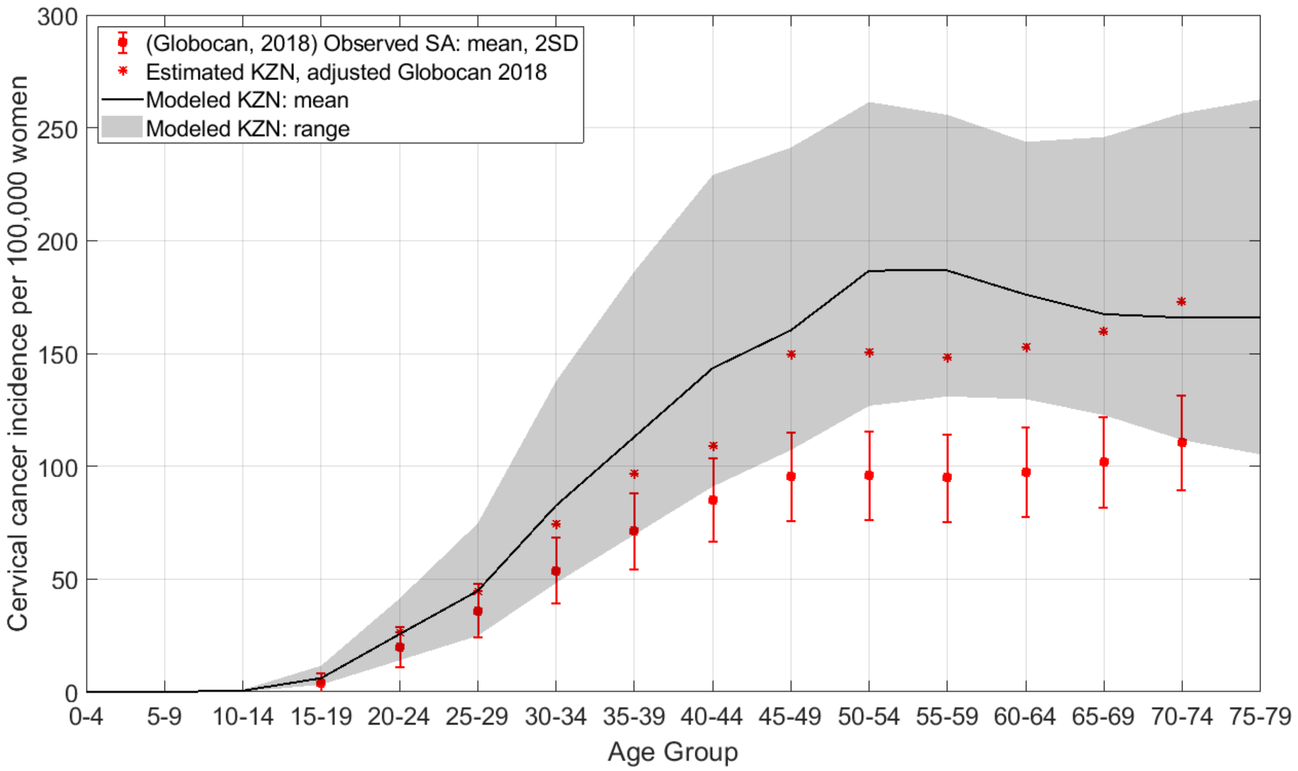

Figure 10. Model fit to observed cervical cancer incidence data by age in 2018. Asterisks represent South Africa Globocan 2018 rates adjusted to take into account higher HIV prevalence in KwaZulu-Natal. Standard deviation of the observed data calculated assuming that incidence follows a normal approximation of the Poisson distribution. Shaded region represents the range of estimates using the 25 best-fitting model parameter sets.

IV. Additional Model Output

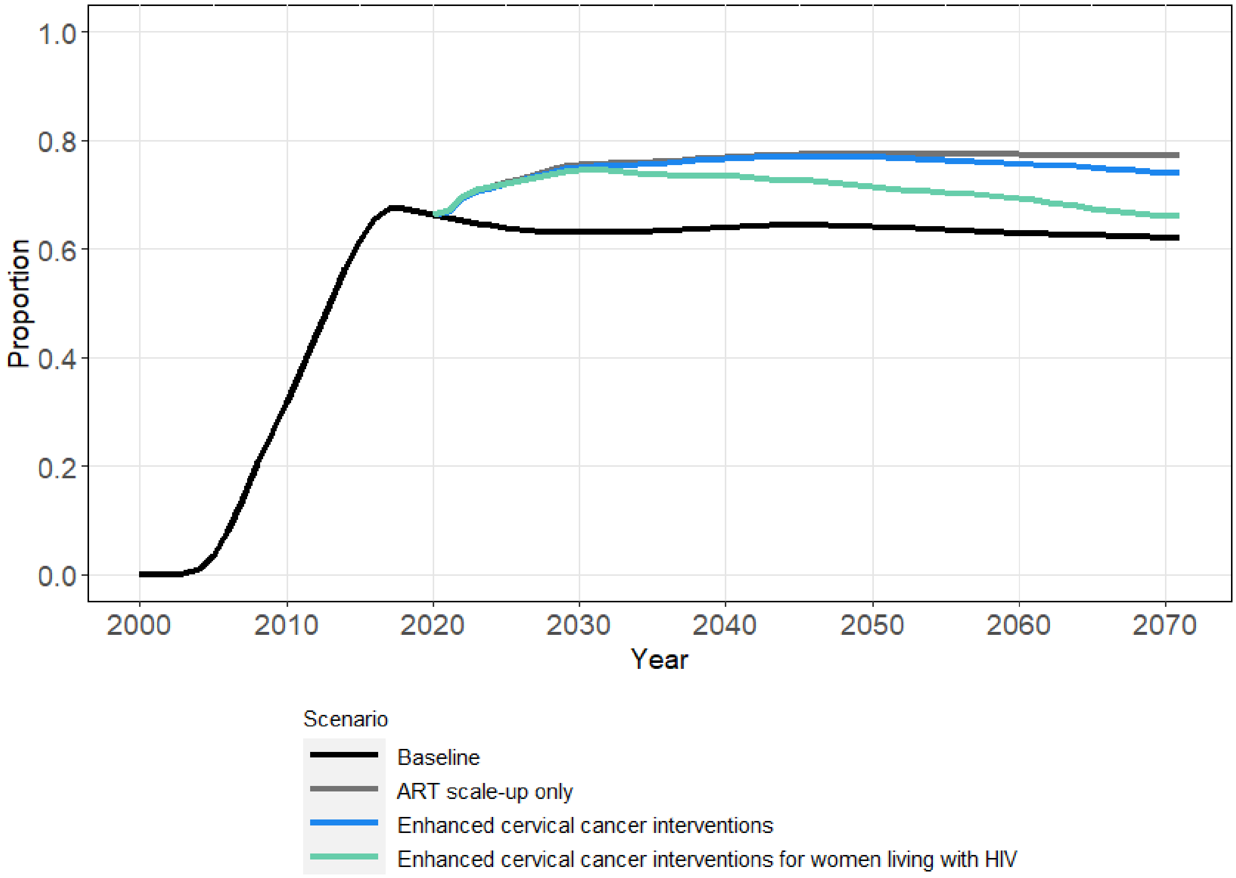

Figure 11. Median proportion of cancer cases among women living with HIV that are in women living with HIV viral suppression under simulated intervention scenarios. Mirroring the introduction of ART in 2004 and observed increase in population-level viral suppression between 2004 to 2017, our model predicted that the proportion of women who are virally suppressed of those living with HIV who develop cervical cancer will also increase. Under the baseline scenario, we projected the proportion of cervical cancer cases among virally suppressed women living with HIV will change from 0.00 [0.00, 0.00] in 2001 (before ART is introduced), to 0.66 [0.61, 0.69] by 2021, and will remain relatively stable with a value of 0.62 [0.54, 0.70] in 2071. In the alternative scenarios, this proportion consistently increased to median values of 0.74-0.76 in 2031. By 2071, the proportion of cases among virally suppressed women living with HIV was 0.77 [0.72, 0.84] in the ART scale-up only scenario, 0.74 [0.65, 0.81] with enhanced cervical cancer interventions, and 0.66 [0.57, 0.75] with enhanced cervical cancer interventions for women living with HIV.

V. References

1. National Department of Health (NDoH) SSASS, South African Medical Research, Council (SAMRC) I. South Africa Demographic and Health Survey 2016. Pretoria, South Africa, and Rockville, Maryland, USA, 2019.
2. Simbayi LC ZK, Zungu N, Moyo S, Marinda E, Jooste S, Mabaso M, Ramlagan S, North A, van Zyl J, Mohlabane N, Dietrich C, Naidoo I, SABSSM V Team. The Fifth South African National HIV Prevalence, Incidence, Behaviour and Communications Survey, 2017. Cape Town, 2019.
3. Tan N, Sharma M, Winer R, Galloway D, Rees H, Barnabas RV. Model-estimated effectiveness of single dose 9-valent HPV vaccination for HIV-positive and HIV-negative females in South Africa. *Vaccine* 2018; **36**(32 Pt A): 4830-6.
4. Rao DW, Bayer CJ, Liu G, et al. Modelling cervical cancer elimination using single-visit screening and treatment strategies in the context of high HIV prevalence: estimates for KwaZulu-Natal, South Africa. *Journal of the International AIDS Society* 2022; **25**(10): e26021.
5. National Department of Health RoSA. National Guideline for Cervical Cancer Control and Management. 2019.
6. Jordaan S, Michelow P, Simoens C, Bogers J. Challenges and Progress of Policies on Cervical Cancer in South Africa. *Health Care : Current Reviews* 2017; **05**(01).
7. National Department of H, Statistics South A, South African Medical Research C, Icf. South Africa Demographic and Health Survey 2016. Pretoria, South Africa and Rockville, Maryland, USA: NDoH, Stats SA, SAMRC, and ICF, 2019.
8. Godfrey MAL, Mathenjwa S, Mayat N. Rural Zulu women's knowledge of and attitudes towards Pap smears and adherence to cervical screening. *Afr J Prim Health Care Fam Med* 2019; **11**(1): e1-e6.
9. Gakidou E, Nordhagen S, Obermeyer Z. Coverage of cervical cancer screening in 57 countries: low average levels and large inequalities. *PLoS Med* 2008; **5**(6): e132.
10. World Health O. WHO estimates of human papillomavirus immunization coverage, 2019 update. World Health Organization; 2019.
11. Kreimer AR, Sampson JN, Porras C, et al. Evaluation of Durability of a Single Dose of the Bivalent HPV Vaccine: The CVT Trial. *J Natl Cancer Inst* 2020; **112**(10): 1038-46.
12. Whitworth HS, Gallagher KE, Howard N, et al. Efficacy and immunogenicity of a single dose of human papillomavirus vaccine compared to no vaccination or standard three and two-dose vaccination regimens: A systematic review of evidence from clinical trials. *Vaccine* 2020; **38**(6): 1302-14.
13. van Schalkwyk C, Moodley J, Welte A, Johnson LF. Modelling the impact of prevention strategies on cervical cancer incidence in South Africa. *International Journal of Cancer* 2021.
14. Maiman M, Tarricone N, Vieira J, Suarez J, Serur E, Boyce JG. Colposcopic evaluation of human immunodeficiency virus-seropositive women. *Obstet Gynecol* 1991; **78**(1): 84-8.
15. Spinillo A, Capuzzo E, Tenti P, De Santolo A, Piazzi G, Iasci A. Adequacy of screening cervical cytology among human immunodeficiency virus-seropositive women. *Gynecol Oncol* 1998; **69**(2): 109-13.
16. Anderson JR, Paramsothy P, Heilig C, et al. Accuracy of Papanicolaou Test among HIV-Infected Women. *Clinical Infectious Diseases* 2006; **42**(4): 562-8.
17. Boardman LA, Peipert JF, Cooper AS, Cu-Uvin S, Flanigan T, Raphael SI. Cytologic-histologic discrepancy in human immunodeficiency virus-positive women referred to a colposcopy clinic. *Obstet Gynecol* 1994; **84**(6): 1016-20.
18. Firnhaber C, Mayisela N, Mao L, et al. Validation of cervical cancer screening methods in HIV positive women from Johannesburg South Africa. *PloS One* 2013; **8**(1): e53494.
19. Arbyn M, Bergeron C, Klinkhamer P, Martin-Hirsch P, Siebers AG, Bulten J. Liquid compared with conventional cervical cytology: a systematic review and meta-analysis. *Obstet Gynecol* 2008; **111**(1): 167-77.

20. Republic of South Africa National Department of H. Cervical Cancer Prevention and Control Policy. 2017.
21. Kelly H, Mayaud P, Segondy M, Pant Pai N, Peeling RW. A systematic review and meta-analysis of studies evaluating the performance of point-of-care tests for human papillomavirus screening. *Sex Transm Infect* 2017; **93**(S4): S36-S45.
22. Johnson LG, Saidu R, Mbulawa Z, et al. Selecting human papillomavirus genotypes to optimize the performance of screening tests among South African women. *Cancer Med* 2020; **9**(18): 6813-24.
23. Saidu R, Kuhn L, Tergas A, et al. Performance of Xpert HPV on Self-collected Vaginal Samples for Cervical Cancer Screening Among Women in South Africa. *J Low Genit Tract Dis* 2020; **25**(1): 15-21.
24. Castle PE, Ajeh R, Dzudie A, et al. A comparison of screening tests for detection of high-grade cervical abnormalities in women living with HIV from Cameroon. *Infect Agent Cancer* 2020; **15**: 45.
25. Chung MH, McKenzie KP, De Vuyst H, et al. Comparing Papanicolau smear, visual inspection with acetic acid and human papillomavirus cervical cancer screening methods among HIV-positive women by immune status and antiretroviral therapy. *AIDS (London, England)* 2013; **27**(18): 2909-19.
26. Magdi R, Elshafeey F, Elshebiny M, et al. A systematic review and meta-analysis of diagnostic accuracy of HPV tests for the screening of cervical cancer in low-resource settings. *International Journal of Gynaecology and Obstetrics: The Official Organ of the International Federation of Gynaecology and Obstetrics* 2020.
27. de Fouw M, Oosting RM, Rutgrink A, Dekkers OM, Peters AAW, Beltman JJ. A systematic review and meta-analysis of thermal coagulation compared with cryotherapy to treat precancerous cervical lesions in low- and middle-income countries. *International Journal of Gynaecology and Obstetrics: The Official Organ of the International Federation of Gynaecology and Obstetrics* 2019; **147**(1): 4-18.
28. Pinder LF, Parham GP, Basu P, et al. Thermal ablation versus cryotherapy or loop excision to treat women positive for cervical precancer on visual inspection with acetic acid test: pilot phase of a randomised controlled trial. *Lancet Oncol* 2020; **21**(1): 175-84.
29. Msyamboza KP, Phiri T, Sichali W, Kwenda W, Kachale F. Cervical cancer screening uptake and challenges in Malawi from 2011 to 2015: retrospective cohort study. *BMC Public Health* 2016; **16**(1): 806.
30. Ouedraogo Y, Furlane G, Fruhauf T, et al. Expanding the Single-Visit Approach for Cervical Cancer Prevention: Successes and Lessons From Burkina Faso. *Glob Health Sci Pract* 2018; **6**(2): 288-97.
31. Blumenthal PD, Gaffikin L, Deganus S, et al. Cervical cancer prevention: safety, acceptability, and feasibility of a single-visit approach in Accra, Ghana. *Am J Obstet Gynecol* 2007; **196**(4): 407.e1-8; discussion .e8-9.
32. Cubie HA, Campbell C. Cervical cancer screening – The challenges of complete pathways of care in low-income countries: Focus on Malawi. *Womens Health (Lond)* 2020; **16**.
33. Shiferaw N, Salvador-Davila G, Kassahun K, et al. The Single-Visit Approach as a Cervical Cancer Prevention Strategy Among Women With HIV in Ethiopia: Successes and Lessons Learned. *Global Health, Science and Practice* 2016; **4**(1): 87-98.
34. World Health O, International Agency for Research on C, African P, Health Research C. Prevention of cervical cancer through screening using visual inspection with acetic acid (VIA) and treatment with cryotherapy. A demonstration project in six African countries: Malawi, Madagascar, Nigeria, Uganda, the United Republic of Tanzania, & Zambia. Geneva: WHO, 2012.
35. Anderson J, Wysong M, Estep D, et al. Evaluation of Cervical Cancer Screening Programs in Côte d'Ivoire, Guyana, and Tanzania: Effect of HIV Status. *PLoS ONE* 2015; **10**(9).
36. Lewis KDC, Sellors JW, Dawa A, Tsu VD, Kidula NA. Report on a cryotherapy service for women with cervical intraepithelial neoplasia in a district hospital in western Kenya. *Afr Health Sci* 2011; **11**(3): 370-6.
37. Oga EA, Brown JP, Brown C, et al. Recurrence of cervical intraepithelial lesions after thermo-coagulation in HIV-positive and HIV-negative Nigerian women. *BMC Womens Health* 2016; **16**.

38. Pfaff C, Singano V, Akello H, et al. Early experiences in integrating cervical cancer screening and treatment into HIV services in Zomba Central Hospital, Malawi. *Malawi Med J* 2018; **30**(3): 211-4.
39. Debeaudrap P, Sobngwi J, Tebeu P-M, Clifford GM. Residual or recurrent precancerous lesions after treatment of cervical lesions in HIV-infected women: a systematic review and meta-analysis of treatment failure. *Clin Infect Dis* 2019.
40. Hoffman SR, Le T, Lockhart A, et al. Patterns of persistent HPV infection after treatment for cervical intraepithelial neoplasia (CIN): A systematic review. *International Journal of Cancer* 2017; **141**(1): 8-23.
41. Khozaim K, Orang'o E, Christoffersen-Deb A, et al. Successes and challenges of establishing a cervical cancer screening and treatment program in western Kenya. *Int J Gynaecol Obstet* 2014; **124**(1): 12-8.
42. Forhan SE, Godfrey CC, Watts DH, Langley CL. A Systematic Review of the Effects of Visual Inspection With Acetic Acid, Cryotherapy, and Loop Electrosurgical Excision Procedures for Cervical Dysplasia in HIV-Infected Women in Low- and Middle-Income Countries. *JAIDS Journal of Acquired Immune Deficiency Syndromes* 2015; **68**: S350.
